# Everybody's Page

**Published:** 1890-08-09

**Authors:** 


### Everybody's Page.

Purchaser: "I understand, Mr. Coldly, that there are bacilli in the ice." Coldly (the ice dealer): "Bacilli? Yes'm, there are. We use the very best of 'em too. Great expense, you know, but we have to please our customers."

In building a hospital for consumption at Melbourne lately, an equal number of beds were provided for men and women. Experience proves this to be a mistaken plan, for the committee find that the proportion of women applying for admittance is only about two-fifths of the number of male applicants.

We are not quite sure what "summer complaint" is, but an old villager gives us the following receipt for blackberry syrup, which is a sure cure for that complaint: Mash the berries. To four quarts of berries put two of hot water, let them stand over night, then strain and add three pounds of sugar. Boil until quite thick, and bottle.

A country doctor, being out for a day's shooting, took his errand-boy to carry the game bag. Entering a field of turnips, the dog pointed, and the boy, overjoyed at the prospect of his master's success, exclaimed, "Oh! master, there's a covey; if you get near 'em, won't you *physic 'em!*"—"Physic them! you young rascal. What do you mean?" said the doctor.—"Why, kill 'em, to be sure," replied the lad.

The usual number of bathing and boating accidents are occurring with the seaside season, chiefly because about half our population seems unable to swim. Nothing is easier for a small child to learn than the art of swimming; send a child regularly to large baths during the summer months, and he will inevitably become a swimmer without any necessity for lessons. Boys and girls should all be taught to swim; the parents that neglect this branch of education have the chance of seeing their children drowned before their eyes while they are sitting on the beach during their autumn holiday.

Tomatoes 2d. a pound. Such is the legend which has greeted us lately on the costers' barrows, and how many of us have taken full advantage of the cheapness of this wholesome and refreshing fruit! Tomatoes, which we are told in the lay press cure jaundice and indigestion and torpid liver, and one half of the ills that flesh is heir to. Certainly tomatoes are excellent feeding, and a clever cook can dish them up, hot and cold, in a different manner for every meal of the week; no table ought to have been without tomatoes all through July. But alas! we have fallen into the evil fashion of only eating forced fruit and vegetables; everything must be out of season that comes to the rich man's table; as though forced fruit ever had the flavour of that which had ripened slowly in the warmth of the sun. Besides, nature has its reasons for giving us rhubarb in the spring, strawberries in the summer, and apples in the autumn. We know better than nature in these days, and eat rhubarb tart in the cold days of February and juiceless apples in the summer. And so our greengrocer's bills are heavy, and our food is only half as wholesome as it should be.

That was a sarcastic philosopher who told an over-anxious mother who wouldn't let her child out in a boat, that it would be wiser not to let the child go to bed, for more people died in their beds than elsewhere.

One medical journal says, "Going to bed on an empty stomach is a good way to invite sleeplessness." A rival journal says, "Eating just before retiring prevents sleep." After which we can only give our readers the wise advice to follow their own inclinations.

For the comfort of people who know what it is to be "flustered," this is the laughable reply of a very bright and accomplished lady teacher who was passing a purely formal examination in physiology: "Where is the alimentary canal?" was demanded. "Really," was the pleasant reply, "I forget whether it is in Indianopolis or Illinois!"

In the thirteenth and fourteenth centuries a leper was not allowed to hold property, was deemed incapable of making a will, and lost all the privileges of citizenship. He was hunted from the towns, and driven from the dwellings of men; he was forbidden to drink from the running stream, lest he should defile it, and it was unlawful for him to touch things that were used for food by man.

The Greeks and Romans in their best days expressed ever the utmost scorn of pain. Epictetus is reported to have told his master if he continued to twist his leg it would break; when that event occurred, instead of any sign of pain Epictetus merely remarked, "I told you so,"—heroic, perhaps, but very irritating. Marcus Aurelius says: "As for pain, if it is intolerable it will quickly dispatch you. If it stays long it is bearable. Your mind in the meantime preserves herself calm by the strength of the opining faculty, and suffers nothing. As for your limbs that are hurt by the pain, if they can complain let them do it."

The fear of death is a feeling we have only in health; when sickness comes death becomes usually a friendly shadow waiting to receive us when our mortal trials become unbearable. The last moments of men are generally unconscious; it is seldom, indeed, that the painful sight of "dying hard," as the poor say, is witnessed. The most trying death-bed ever attended by the writer, who has watched by hundreds, was that of a youth of eighteen, dying of acute pneumonia. For the last twenty-four hours he was conscious, but partly delirious, and he lay gasping for breath and ejaculating prayers to the effect that God would save his father and mother. The painful part was that his father and mother were thoroughly decent, sober, Church-going folk, and it jarred on every sense of fitness that this poor boy should imagine himself sure of salvation, and his parents still in an evil state. If religious mania had not from the first led this youth to dwell on death he might possibly have recovered, but he made no fight for life; told the doctor he was "saved," he did not fear to die. Here was a case where fear of death might have had a salutary effect.

